# βPS-Integrin acts downstream of Innexin 2 in modulating stretched cell morphogenesis in the *Drosophila* ovary

**DOI:** 10.1093/g3journal/jkab215

**Published:** 2021-06-28

**Authors:** Yi-Chia Huang, Kuan-Han Chen, Yu-Yang Chen, Liang-Hsuan Tsao, Tsung‐Han Yeh, Yu-Chia Chen, Ping-Yen Wu, Tsu-Wei Wang, Jenn-Yah Yu

**Affiliations:** 1 Department of Life Sciences and Institute of Genome Sciences, National Yang Ming Chiao Tung University, Taipei 112, Taiwan; 2 Department of Life Science, National Taiwan Normal University, Taipei 116, Taiwan; 3 Brain Research Center, National Yang Ming Chiao Tung University, Taipei 112, Taiwan

**Keywords:** Innexin, integrin, microtubule, oogenesis, morphogenesis, stretched cells, follicle cells

## Abstract

During oogenesis, a group of specialized follicle cells, known as stretched cells (StCs), flatten drastically from cuboidal to squamous shape. While morphogenesis of epithelia is critical for organogenesis, genes and signaling pathways involved in this process remain to be revealed. In addition to formation of gap junctions for intercellular exchange of small molecules, gap junction proteins form channels or act as adaptor proteins to regulate various cellular behaviors. In invertebrates, gap junction proteins are Innexins. Knockdown of *Innexin 2* but not other *Innexin*s expressed in follicle cells attenuates StC morphogenesis. Interestingly, blocking of gap junctions with an inhibitor carbenoxolone does not affect StC morphogenesis, suggesting that Innexin 2 might control StCs flattening in a gap-junction-independent manner. An excessive level of βPS-Integrin encoded by *myospheroid* is detected in *Innexin 2* mutant cells specifically during StC morphogenesis. Simultaneous knockdown of *Innexin 2* and *myospheroid* partially rescues the morphogenetic defect resulted from *Innexin 2* knockdown. Furthermore, reduction of βPS-Integrin is sufficient to induce early StCs flattening. Taken together, our data suggest that βPS-Integrin acts downstream of Innexin 2 in modulating StCs morphogenesis.

## Introduction

Epithelial morphogenesis is key to construct developing tissues and organs. During morphogenesis, communication through signal transduction or direct intercellular connection is required for the coordination of cell shape change and rearrangement. Gap junctions form direct intercellular connection, which is an effective way for synchronizing cells such as neurons and cardiomyocytes (Rohr 2004; Sohl *et al.* 2005). In addition, gap junction proteins are essential for pattern formation during development (Levin 2007). However, the underlying mechanisms of morphogenesis regulated by gap junction proteins remain to be investigated.

Gap junctions are composed of juxtaposed channels on adjacent cells. These juxtaposed channels are formed by Connexins (Cxs) in vertebrates and Innexins (Inxs) in invertebrates. Six Cxs constitute a Connexon; and two Connexons form a gap junction (Goodenough *et al.* 1996; Revel *et al*. 1985). Inxs, on the other hand, may form hexadecameric gap junctions (Oshima *et al.* 2016). Gap junctions allow connected cells to transmit small molecules and metabolites such as calcium, inositol phosphates, and cyclic nucleotides (Nielsen *et al.* 2012). In addition, gap junction proteins interact with other proteins to regulate cell adhesion or cell polarity independent of their gap junction properties (Bauer *et al.* 2004; Elias *et al.* 2007; Giuliani *et al.* 2013; Ghezali *et al.* 2018). Thus, it is important to unravel functions or mechanisms of gap junction proteins in modulating various biological processes.

In *Drosophila*, there are eight *Inx* genes playing diverse roles in different tissues (Bauer *et al.* 2005; Phelan 2005). Many finding suggest that Inxs have critical functions in the neuronal circuit of the nervous system, including Inx6, Inx7, and Shaking B (ShakB or Inx8) (Phelan *et al.* 1996; Sun and Wyman 1996; Phelan *et al.* 1998; Wu *et al.* 2011). Furthermore, mutation of *optic ganglion reduced* (*ogre* or *Inx1*) causes smaller optic lobes due to reduction of postembryonic neuroblasts (Lipshitz and Kankel 1985). Gap junctions composed of Inx1 and Inx2 in glial cells of the blood-brain barrier coordinate calcium oscillations in response to nutritional signals and control secretion of insulin (Holcroft *et al.* 2013; Speder and Brand 2014). In addition, gap junction proteins are important for epithelial morphogenesis. Inx2 and Inx3 regulate epithelial organization and polarity in the embryonic epidermis (Lehmann *et al.* 2006). Inx3 is required for dorsal closure, a morphogenetic event during mid-embryogenesis (Giuliani *et al.* 2013). *Inx2* is a target gene of the Wingless pathway mediating morphogenetic movements during gut organogenesis (Bauer *et al.* 2002). In the embryonic epithelia, Inx2 directly interacts with *Drosophila* E-Cadherin (DE-cad) and Armadillo (Arm), demonstrating that Inx2 regulates cell polarity through protein–protein interaction with components of cellular junctions (Bauer *et al.* 2004).

To investigate functions of gap junction proteins, the *Drosophila* ovary provides a convenient model (Rubin and Huynh 2015). The *Drosophila* ovary is composed of 15–20 ovarioles containing egg chambers at different stages. Based on morphological characters and cell cycle status, oogenesis is categorized into 14 stages (Jia *et al.* 2016). The germarium located at the anterior tip of the ovariole contains both germline and follicle stem cells (FSCs), which divide and generate egg chambers with 16-cell germline cysts enwrapped by a monolayer of follicular epithelium. Egg chambers move posteriorly and develop into eggs (Bastock and St Johnston 2008). Prior to stage 6, follicle cells undergo mitosis and produce about 650 cells. At stage 7, the Notch pathway drives the switch from mitosis to endocycle (Deng *et al.* 2001). From stage 9 to 10, follicle cells reorganize through a series of migratory and morphogenetic behaviors. Six to eight anterior-most follicle cells are specified into border cells and migrate with the anterior polar cells toward the border between nurse cells and the oocyte. About 50 anterior follicle cells are specified into stretched cells (StCs) and flatten drastically to cover nurse cells. At the same time, posterior follicle cells form a layer of columnar epithelium surrounding the oocyte (Wu *et al.* 2008).

Several genes and signaling pathways that regulate morphogenesis of StCs have been identified. Results from a quantitative morphometric analysis suggest that morphogenesis of StCs may be passively caused by enlargement of germline cells (Kolahi *et al.* 2009). Another study suggests that the Transforming Growth Factor β (TGF-β) pathway promotes StC flattening via activating the Notch pathway and remodeling of the adherens junctions (Brigaud *et al.* 2015), suggesting that StC morphogenesis is active. Hindsight (Hnt), a Zinc-finger containing transcription factor, modulates StC morphogenesis by down-regulating adhesion molecules, such as Arm, DE-cad, DN-cad, and Fasciclin 3 (Fas3) (Melani *et al.* 2008). A Ser/Thr kinase Tao modulates StC flattening by promoting endocytosis of Fasciclin 2 (Fas2) at the lateral membrane prior to StC morphogenesis (Gomez *et al.* 2012). In addition, adhesion molecules such as integrins are critical for cell morphogenesis (Daley and Yamada 2013). Integrins are αβ heterodimeric cell surface receptors connecting the extracellular matrix to the cytoskeleton (Bulgakova *et al.* 2012). In *Drosophila*, there are two β subunits, βPS and βn, and five α subunits of integrins. βPS encoded by *myospheroid* (*mys*) is the only β subunit detected in the ovary (Fernandez-Minan *et al.* 2008). However, roles of integrins in StC morphogenesis have not been carefully examined (Chlasta *et al.* 2017).

Gap junction proteins have been shown to play various roles during oogenesis. *zero population growth* (*zpg* or *Inx4*) is required for the differentiation of both male and female germlines (Gilboa *et al.* 2003; Smendziuk *et al.* 2015). Microinjection of anti-Inx2 antibodies into the oocyte stops oogenesis (Bohrmann and Zimmermann 2008). A mutant allele of *Inx2* leads to aberrant cyst and egg chamber formation (Mukai *et al.* 2011). Inx2 is also required for border cell induction through modulating calcium flux between follicle cells, which in turn promotes endocytosis and activation of the JAK/STAT pathway (Sahu *et al.* 2017). A recent study demonstrates that Inx2 and Inx3 regulate microtubules in border cells, which is critical for border cells to integrate into the epithelium upon arrival at the oocyte (Miao *et al.* 2020). Interestingly, functions of channels or gap junctions are dispensable for this neolamination process of border cells, suggesting various roles and molecular mechanisms of gap junction proteins.

To identify roles of Inxs, we tested four *Inx* genes expressed in follicle cells, including *ogre* (*Inx1*), *Inx2*, *Inx3*, and *Inx7* (Stebbings *et al.* 2002). Only *Inx2* was required for regulating StC morphogenesis. Blocking of gap junction functions did not affect StC morphogenesis, suggesting that Inx2 may promote StC flattening in a gap junction-independent manner. The level of βPS encoded by *mys* was significantly higher in *Inx2* loss-of-function StCs than that in control cells. Importantly, simultaneous knockdown of *Inx2* and *mys* partially rescued the morphogenetic defects of StCs caused by *Inx2* knockdown. Reduction of βPS induced early StC flattening. These results suggest that βPS acts downstream of Inx2 in modulating StC flattening. Interestingly, the level of microtubules was increased in *Inx2* mutant cells. While microtubules are critical for integrin trafficking, this may provide a mechanism for regulation of integrins by Inx2 (Seetharaman and Etienne-Manneville 2019). Taken together, we demonstrate a novel genetic interaction between *Inx2* and *mys* in modulating cell morphogenesis, possibly through a gap junction-independent mechanism.

## Materials and methods

### Fly strains

The following *Drosophila* strains were used for RNAi knockdown experiments:

*Inx1* (*ogre*)*: y^1^ v^1^; P{TRiP.JF02595}attP2* (*BLM 27283*)*, Inx2*: *y^1^ v^1^; P{TRiP.JF02446}attP2* (*BLM 29306*), *Inx3*: *w1118; P{GD14965}v39094*, *y^1^ v^1^; Inx7: P{TRiP.JF02066}attP2* (*BLM26297*), *mys*: *w1118; P{GD15002}*(*v29619*)*, mys: P{KK100518}VIE-260B* (*v103704*) *UAS-GFP. GAL4* lines: *P{GawB}c306*

The following *Drosophila* strains were used for generation of mitotic clones:

P{neoFRT}19A, ey−flp

$$w^{67c23}P\left\{\mathrm{lacW}\right\}Inx2^{G0059}P\left\{\mathrm{neoFRT}\right\}19A/FM7c;\;P\left\{ey-\mathrm{FLP}.N\right\}5$$

$$w^{67c23}P\left\{\mathrm{lacW}\right\}Inx2^{G0157}P\left\{\mathrm{neoFRT}\right\}19A/FM7c;\;P\left\{ey-\mathrm{FLP}.N\right\}5$$

$$y^{1}w*\;\mathrm{Inx}2^{B}P\left\{\mathrm{neoFRT}\right\}19A/FM7c,\;P\left\{\mathrm{GAL}4-Kr.C\right\}DC1,\;P\left\{\mathrm{UAS}-\mathrm{GFP}.S65T\right\}DC5,\;sn^{+}$$

$$y^{1}w*\;\mathrm{Inx}2^{A}P\left\{\mathrm{neoFRT}\right\}19A/FM7c,\;P\left\{\mathrm{GAL}4-Kr.C\right\}DC1,\;P\left\{\mathrm{UAS}-\mathrm{GFP}.S65T\right\}DC5,\;sn^{+}.$$

$$P\left\{\mathrm{hsFLP}\right\}1,\;P\left\{\mathrm{tubP}-\mathrm{GAL}80\right\}LL1\;w^{*}P\left\{\mathrm{neoFRT}\right\}19A;\;Pin^{Yt}/\mathrm{CyO}$$

### Generation of mosaic analysis with a repressible cell marker clones

The FLP/FRP site-specific recombination system was applied to generate homozygous GFP-positive mutant clones with a heatshock promoter (Wu and Luo 2006). Newly eclosed flies were collected for heatshock at 37°C for four times in constitutively 2 days. On the first day, flies were heatshocked twice for 30 minutes with a 3-hours interval between heatshocks; on the second day, flies were heatshocked once for 30 minutes and once for 60 minutes with a 3-hours interval between heatshocks. All flies were cultured at 25°C for 6 days before dissection.

### RNAi experiments

Flies were raised at 18°C before eclosion. Newly eclosed adult flies were collected and grown at 29°C for 6 days before dissection.

### Egg chambers culturing and application of Gap junction blockers

*Drosophila* ovaries were dissected in Schneider’s medium containing 15% fetal bovine serum, 0.6× penicillin/streptomycin, and 0.10 mg/ml insulin (Prasad *et al.* 2007). Medium was maintained at 25°C and replaced every 1 hour and a half. Carbenoxolone was dissolved in water and used at 0.2 mM (Speder and Brand 2014). Ovaries were fixed as described below.

### Immunofluorescence staining and microscopy

Ovaries were dissected in phosphate-buffered saline (PBS) and fixed in 4% paraformaldehyde (PFA) in PBS for 15 minutes. After fixation, ovaries were washed with PBT (1XPBS, 0.5% Triton X-100) for 3 times. Next, ovaries were incubated in the blocking solution PBTB (1XPBS, 0.5% Triton X-100, 5% goat serum, 2.5 mg/ml BSA, and 0.05% Sodium azide) at room temperature followed by incubation in PBTB with the primary antibodies for overnight at 4°C. Ovaries were then washed with PBT and incubated with secondary antibodies for overnight at 4°C. The following antibodies were used: mouse anti-Armadillo (1:200 dilution, N2 7A1 Armadillo, DSHB), mouse anti-Discs large (1:200, 4F3 anti-discs large, DSHB), mouse anti-Integrin βPS (1:200, CF.6G11, DSHB), mouse anti-Cut (1:200, 2B10, DSHB), mouse anti-Hindsight (1:200, 1G9, DSHB), mouse anti-Fasciclin II (1:200, 1D4 anti-Fasciclin II), rat anti-DE-cadherin, extracellular domain (1:200, DCAD2, DSHB), rabbit anti-GFP (1:1000, Invitrogen), guinea pig anti-Inx2 (1:1000, provided by Dr. Guy Tanentzapf), anti- α Tubulin (1:1000, DM1A, Sigma-Aldrich), anti-β Tubulin (1:200, AA12.1, DSHB), Dylight-488 goat anti-rabbit IgG (H + L), Dylight-549 goat anti-mouse IgG (H + L), Dylight-549 goat anti-guinea pig IgG (H + L) (Jackson ImmunoReasearch Laboratories), and Alexa Fluor 633 goat anti-mouse IgG (H + L) (Invitrogen). Ovaries were further stained with DAPI in PBT (1 μg/ml, Sigma) prior to mounting with mounting solution [85% glycerol, 1XPBS, 3% propyl gallate (Sigma), and Prolong^®^ Gold Antifade reagents (Invitrogen, Carlsbad, CA, USA)]. All images were taken by using Zeiss LSM700 (Carl Zeiss AG, Germany). The images were further arranged and optimized by using Adobe Photoshop CS3 (San Jose, CA, USA).

### Images analysis and quantification

Single slice of confocal images were used for analysis of the fluorescence intensities. The fluorescence intensities were measured by using ImageJ (NIH, USA). The value of “Mean Intensity” in ImageJ was used. One to three *Inx2* mutant cells and adjacent control cells of the same picture were selected for measurement. At least seven images with marked clones were measured for each genotype. The relative fluorescence intensities in the apical region of DE‐cad, Arm, or βPS, and the lateral region of α and β Tubulin, were calculated. Stages 9 and 10 egg chambers were selected for measurement of the distance between StC nuclei in a single confocal plane. The distance between adjacent StC nuclei was measured and calculated by using the line tool in Zen (blue edition, Zeiss, Germany). For quantification of the fluorescent intensity of *stat::*GFP, egg chambers at stage 8 were selected. Those border cells adjacent to the anterior polar cells with stronger GFP intensities in a confocal plane were selected for measurement. The mean intensity of the cytoplasmic region of the individual cell was measured. The mean intensity of the cytoplasmic region of polar cells was used as background for subtraction.

### Proximity ligation assay

Ovaries were dissected in PBS, fixed in 4% PFA in PBS and followed by immunofluorescence staining protocol for incubation of primary antibodies. After washing, the manufacturer's protocol (Sigma-Aldrich/Thermo Fisher USA) modified for whole-mount tissue staining was followed (Wang *et al.* 2015). Goat anti-guinea pig IgG (Invitrogen, USA) was conjugated with Plus oligonucleotide following the manufacturer's protocol (Duolink *in situ* Probemaker Plus, Sigma-Aldrich/Thermo Fisher USA). Single slice of confocal image was used. For quantification of proximity ligation assay (PLA) data, punctate staining signals were counted.

## Results

### *Inx2* is required for StC morphogenesis

**Figure 1:**
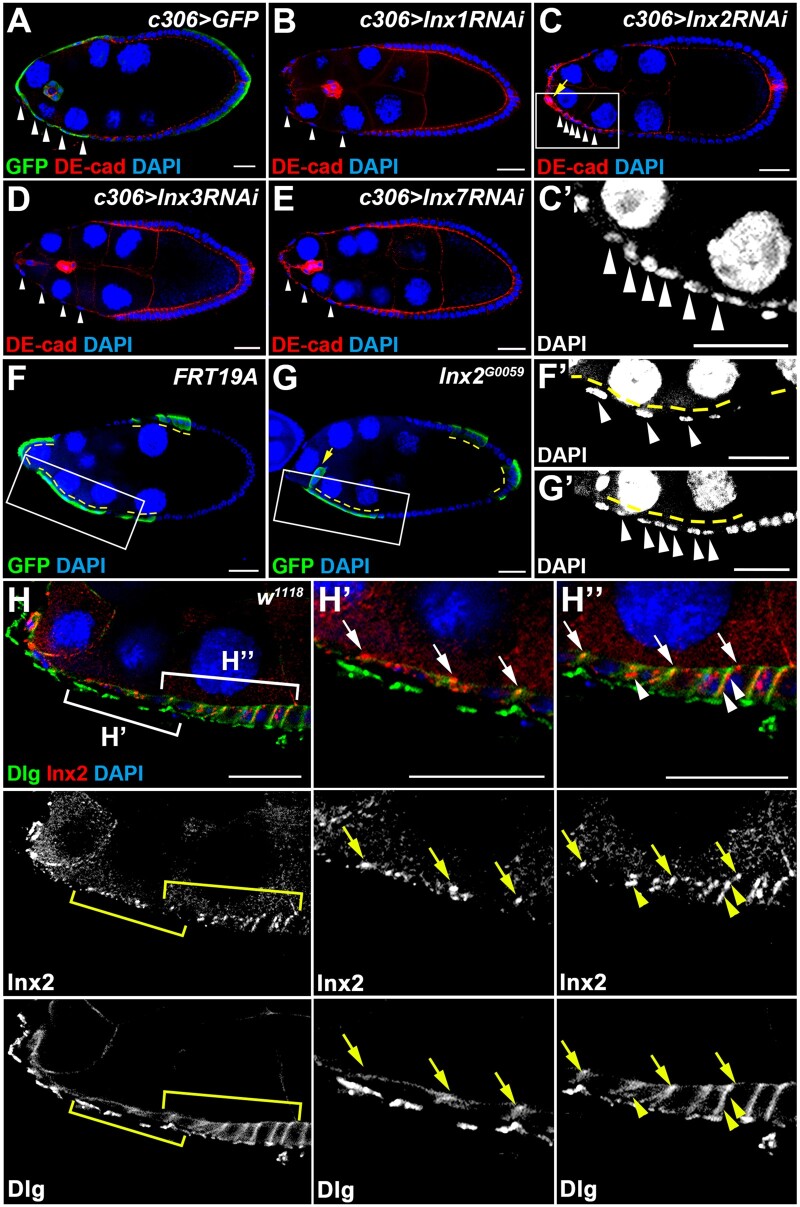
Inx2 is required for StC morphogenesis. Egg chambers at stage 9 were selected and oriented as anterior to the left. Ovaries were stained with anti-DE-cad (A–E), anti-GFP (A, F, and G), anti-Dlg (H), anti-Inx2 (H), and DAPI for DNA in cell nuclei. High magnification views are shown in (C’, F’, G’, H’, and H’’). (A–E) Newly eclosed flies were grown at 29°C for 6 days before dissection. *UAS-RNAi* targeting *Inx*s were driven by *c306-GAL4*. *UAS-GFP* driven by *c306-GAL4* was used as a control. StC nuclei are indicated by white arrowheads. (A, B, D, and E) In control, *Inx1*, *Inx3*, and *Inx7* knockdown groups, StCs became squamous and their nuclei were separated from one another. (C, C’) In *Inx2* knockdown group, StCs were not flattened and their nuclei were close to one another. The border cell cluster failed to form (the yellow arrow). (F, F’, G, and G’) GFP-positive *FRT19A* or *Inx2^G0059^* mutant clones (yellow dashed lines) were generated by using MARCM and examined 6 days after clone induction. StC nuclei are indicated by white arrowheads. (F) In *FRT19A* control, nuclei of StCs were sparsely separated from one another. (G) Nuclei of *Inx2* mutant StCs were close to one another. The border cell cluster failed to detach from StCs (the yellow arrow). (H) White brackets indicate the regions shown in (H’) and (H’’). Inx2 was detected on both apical membrane (arrows) and lateral membrane (arrowheads) of StCs during morphogenesis. Length of the scale bar is 20 μm.

mRNAs of four gap junction protein genes, *ogre* (*Inx1*)*, Inx2, Inx3*, and *Inx7*, are detected in follicle cells during oogenesis (Stebbings *et al.* 2002). To investigate roles of gap junction proteins in follicle cells, we used *c306-GAL4* to drive expression of shRNA in follicle cell precursors, anterior/posterior follicle cells, polar cells, StCs, and border cells (Supplementary Figure S1). *UAS-GFP* driven by *c306-GAL4* was used as a control. Immunofluorescence of DE-cad was used for labeling the apical-lateral membrane of follicle cells and border cell clusters (Geisbrecht and Montell 2002; Yeh *et al.* 2015). Knockdown of *Inx*s driven by *c306-GAL4* did not affect follicle cell differentiation during early oogenesis (data not shown). During StC flattening at stage 9, the distance between StC nuclei was increased dramatically as shown in the *UAS-GFP* control and *Inx1*, *Inx3*, and *Inx7* knockdown groups (Figure 1, A, B, D, and E). When *Inx2* was knocked down, StCs nuclei were distributed close to one another (Figure 1C, C’, Supplementary Table S1), suggesting attenuation of StC morphogenesis. Inx2 has been reported to regulate border cell fate by controlling the JAK/STAT signaling (Sahu *et al.* 2017). Consistently, knockdown of *Inx2* driven by *c306-GAL4* resulted in defective border cell migration (Figure 1C and data not shown). To confirm the phenotype of *Inx2* loss-of-function, we used mosaic analysis with a repressible cell marker (MARCM) to generate *Inx2* mutant clones (Wu and Luo 2006). As a control, *FRT19A* GFP-positive StCs underwent cell flattening and their cell nuclei were sparsely distributed at stage 9 (Figure 1, F and F’). The cell nuclei of GFP-positive *Inx2* mutant StCs remained tightly distributed (Figure 1, G and G’). *Inx2* mutant border cells formed a cluster successfully but failed to detach from StCs (Figure 1G), demonstrating that those tightly distributed *Inx2* mutant cells surrounding the nurse cells are StCs that failed to flatten, but not border cells. Taken together, these data show that *Inx2* is required for StC morphogenesis.

### Inx2 is distributed at both apical and lateral membrane of follicle cells

We next examined the distribution of Inx2 in the ovary by using immunofluorescence. Inx2 was not detected in GFP-positive *Inx2* mutant cells (Supplementary Figure S2A), demonstrating the specificity of anti-Inx2 antibody (anti-Inx2) (Smendziuk *et al.* 2015). Immunofluorescence of Disc large-1 (Dlg) was used for labeling the lateral membrane of follicle cells (Yeh *et al.* 2015). Inx2 was detected on the membrane between germline cells (Supplementary Figure S2, B, C, and C’). In follicle cells, Inx2 was mainly detected on the apical membrane during early oogenesis (Supplementary Figure S2, B, C, and C’). At stage 9, puncta of anti-Inx2 staining were observed at both apical and lateral membrane of follicle cells (Figure 1, H, H’, and H’’), demonstrating that Inx2 is detected in StCs.

### Blocking of gap junctions with a gap junction blocker carbenoxolone is not sufficient to attenuate StC morphogenesis

**Figure 2:**
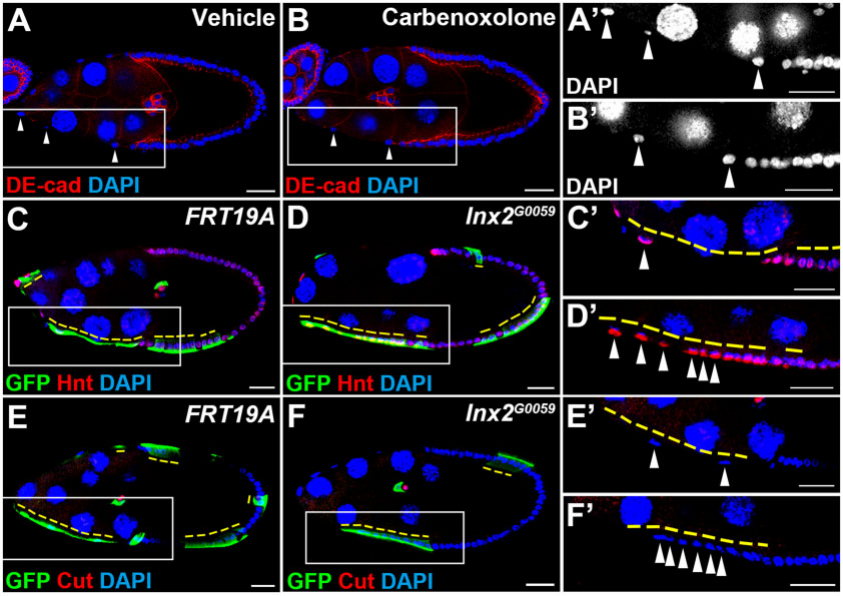
Blocking of gap junction activity is not sufficient to attenuate StC morphogenesis and Inx2 is not required for the mitosis-to-endocycle transition. Egg chambers at stage 9 were selected and oriented as anterior to the left. Ovaries were stained with anti-DE-cad (A, B), anti-GFP (C–F), anti-Hnt (C, D), anti-Cut (E, F), and DAPI. High magnification views are shown in (A’–F’). StC nuclei are indicated by white arrowheads. (A, B) Freshly dissected ovaries from *w^1118^* were cultured in Schneider’s medium with vehicle control or carbenoxolone (0.2 mM) for 3 hours. StC nuclei were distributed sparsely in both groups. (C–F) GFP-positive *FRT19A* or *Inx2^G0059^* mutant clones (yellow dashed lines) were generated by using MARCM and examined 6 days after clone induction. (C, E) In *FRT19A* control, StCs became squamous and their nuclei were distributed sparsely. Hnt was detected in StCs, border cells, and main body follicle cells. Cut was detected in polar cells. (D, F) *Inx2* mutant StCs failed to flatten and remained positive for Hnt and negative for Cut as those adjacent GFP-negative control cells. Length of the scale bar is 20 μm.

Since Inx2 is detected on both the apical and lateral membrane of follicle cells (Figure 1H), Inx2 may form gap junctions for intercellular communication between follicle and germline cells and/or among follicle cells. Thus, we examined whether the gap junction activity was required for StC morphogenesis by using a gap junction/channel blocker carbenoxolone. Carbenoxolone has been applied to block gap junctions in the nervous system and ovary of *Drosophila* successfully (Speder and Brand 2014; Sahu *et al.* 2017; Miao *et al.* 2020). It takes approximately 6 hours for StC morphogenesis from stages 9 and 10 (Horne-Badovinac and Bilder 2005), so we cultured ovaries from wild-type flies for 3 or 6 hours in Schneider medium containing either carbenoxolone or vehicle control and examined whether StC morphogenesis was affected. Unexpectedly, incubation in carbenoxolone did not change the distribution of StC cell nuclei comparing with that of the vehicle control at stages 9 and 10 (3 hours in Figure 2, A and B and 6 hours in Supplementary Figure S3, A and B). To test the effectiveness of carbenoxolone treatment, we examined the fluorescent intensity of *stat*::GFP, a reporter for the JAK/STAT pathway. The immunofluorescent intensity of *stat*::GFP has been shown to be regulated by gap junction formation of Inx2 (Sahu *et al.* 2017). After incubation in 200 μM carbenoxolone for 4 hours, the *stat*::GFP immunofluorescent intensity was significantly reduced in border cells (Supplementary Figure S3, C–F), demonstrating the effectiveness of carbenoxolone treatment in suppressing gap junction functions in our *ex vivo* culture system. Thus, in addition to gap junction formation, Inx2 might modulate StC morphogenesis through a gap junction-independent mechanism.

### Defective StC morphogenesis in *Inx2* mutants is not caused by delay of follicle cell differentiation

Defective StC morphogenesis may be caused by delay of follicle cell maturation, which subsequently leads to postponement of StC morphogenesis. Prior to StC morphogenesis during stage 6 to 7, the Notch pathway regulates the mitosis to endocycle (M/E) transition of follicle cells through up-regulation of a zinc-finger transcription factor Hindsight (Hnt) and down-regulation of a homeodomain protein Cut (Deng *et al.* 2001; Lopez-Schier and St Johnston 2001; Sun and Deng 2005, 2007). To exclude the possibility that maturation of *Inx2* mutant follicle cells is delayed, we examined the M/E transition of *Inx2* mutant cells. In *FRT19A* control at stage 9, Hnt was detected in StCs, border cells and posterior follicle cells; Cut was only detected in polar cells (Figure 2, C, C’, E, and E’). Despite failure of morphogenesis, *Inx2* mutant StCs was positive for Hnt and negative for Cut as that of the adjacent control StCs (Figure 2, D, D’, F, and F’), demonstrating that Inx2 mutant cells undergo M/E transition normally. Therefore, it is unlikely that the defect of morphogenesis in *Inx2* mutant StCs is caused by delay of follicle cell maturation.

### The TGF-β pathway may not act downstream of Inx2 in regulating StC morphogenesis

A previous study has demonstrated that the TGF-β pathway promotes StC morphogenesis through remodeling of adherens junctions and cytoskeletons (Brigaud *et al.* 2015). To test whether Inx2 interacts with the TGF-β pathway during StC morphogenesis, we examined the activity of the TGF-β pathway by a *Dad-lacZ* (*Dad*, *Daughter against dpp*) enhancer trap line. The TGF-β pathway is activated in germline stem cells and follicle cells during StC morphogenesis and centripetal migrating cells at stage 10B (Kai and Spradling 2003). The expression of *Dad-lacZ* was barely detected in *Inx2* mutant FSCs and follicle cells as well as adjacent control cells in the germarium at stage 2 (Supplementary Figure S4A). Interestingly, *Dad-lacZ* was up-regulated from stages 4 to 7 in *Inx2* mutant follicle cells comparing with the GFP-negative neighboring control cells (Supplementary Figure S4, B and C). Strong expression of *Dad-lacZ* was detected in *Inx2* mutant StCs and main body follicle cells at stage 10 (Supplementary Figure S4D). Since activation of the TGF-β pathway promotes StC morphogenesis (Brigaud *et al.* 2015), it is unlikely that Inx2 promotes StC morphogenesis through inhibiting the TGF-β pathway.

### Inx2 regulates βPS in StCs during morphogenesis

**Figure 3:**
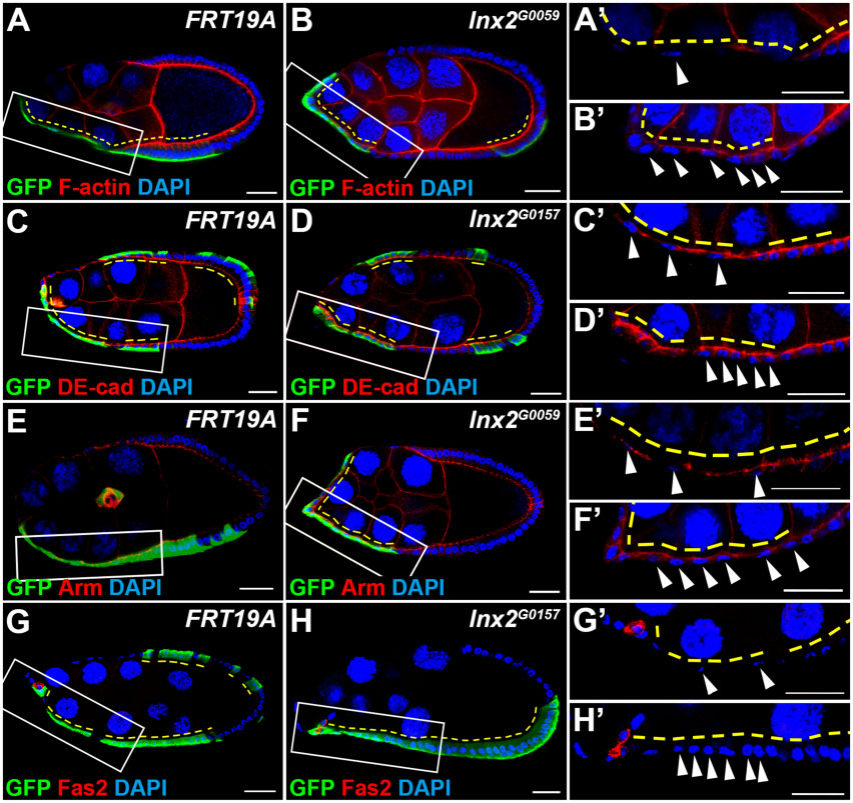
Inx2 does not regulate the actin cytoskeleton, Arm, and Fas2 during StC morphogenesis. Egg chambers at stage 9 were selected and oriented as anterior to the left. GFP-positive *FRT19A*, *Inx2^G0059^* or *Inx2^G0157^* mutant clones (yellow dashed lines) were generated by using MARCM and examined 6 days after clone induction. Ovaries were stained with anti-GFP (A–H), phalloidin (A, B), anti-DE-cad (C, D), anti-Arm (E, F), anti-Fas2 (G, H), and DAPI. (A’–H’). High magnification views are shown in the right panels. StC nuclei are indicated by white arrowheads. (A, A’, C, C’, E, E’, G, and G’) F-actin, DE-cad, Arm, and Fas2 were detected in *FRT19A* control StCs. (B, B’, F, F’, H, and H’). The intensity and distribution of staining signals for F-actin, Arm, and Fas2 in *Inx2* mutant StCs were similar to the adjacent GFP-negative controls. (D, D’) The intensity of staining signals for DE-cad in *Inx2* mutant StCs was slightly increased comparing to the adjacent GFP-negative controls. Length of the scale bar is 20 μm.

We next examined whether Inx2 modulates StC morphogenesis through regulating actin cytoskeletal rearrangement, cellular junctions or cell adhesion. *FRT19A* and *Inx2* mutant MARCM clones were generated and stained for actin cytoskeleton or components of junctional and adhesion complexes. Single slice of confocal images were used for image analysis. Neither the level nor the distribution of filamentous actin was changed in GFP-positive *Inx2* mutant cells in comparison with *FRT19A* control clones or neighboring GFP-negative control cells (Figure 3, A and B), suggesting that Inx2 does not regulate StC morphogenesis through rearrangement of the actin cytoskeleton. It has been reported that Inx2 is required for epithelial morphogenesis in the *Drosophila* embryo through interaction with components of adherens junction complex, such as DE-cad and Arm (Bauer *et al.* 2004). In addition, the Ser/Thr kinase Tao promotes endocytosis of a cell adhesion molecule Fas2 on the lateral domain to relieve intercellular connection and facilitate StC morphogenesis (Gomez *et al.* 2012). Thus, we examined the level and distribution of DE-cad, Arm, and Fas2. In *FRT19A* control clones at stage 9, DE-cad or Arm was detected on the apical-lateral domain of follicle cells and StCs (Figure 3, C, C’, E, and E’). The immunofluorescent intensity of DE-cad in GFP-positive *Inx2* mutant cells were slightly increased comparing with the neighboring GFP-negative control cells (Figure 3, D and D’, Supplementary Figure S5). The level and distribution of Arm in *Inx2* mutant cells were not significantly different from that of the neighboring GFP-negative control cells (Figure 3, F and F’, Supplementary Figure S5). Fas2 was enriched in polar cells and barely detected in *FRT19A* control StCs at stage 9 (Figure 3, G and G’). The level and distribution of Fas2 in *Inx2* mutant cells were similar to that of the neighboring GFP-negative control cells (Figure 3, H and H’). Taken together, Inx2 may not regulate StC flattening through modulating actin cytoskeleton, Arm, or Fas2-mediated lateral adhesion. Inx2 may regulate StC morphogenesis through modulating DE-cad. Since the fold-change of DE-cad immunofluorescent intensity in *Inx2* mutant cells is only 1.33 fold (Supplementary Figure S5), this regulation might not be the primary mechanism for the StC morphogenetic defect in *Inx2* mutant cells.

**Figure 4:**
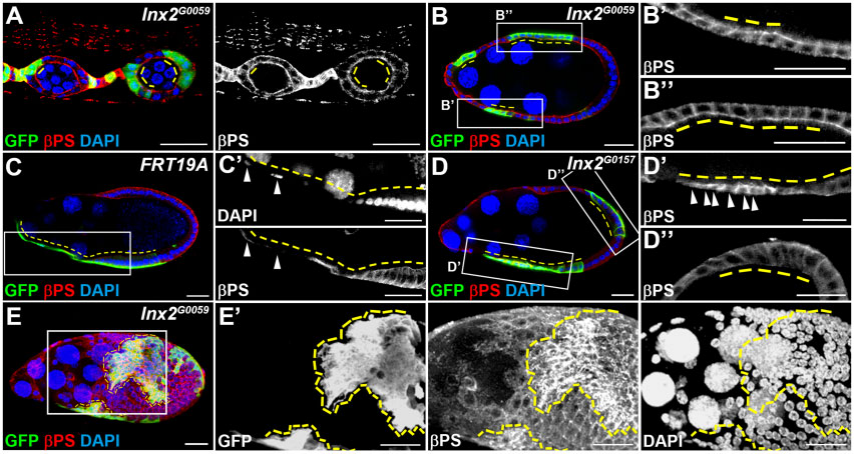
Inx2 modulates the level of and distribution of βPS specifically in StCs. Egg chambers at different stages were selected and oriented as anterior to the left. GFP-positive *FRT19A* and *Inx2^G0059^* mutant clones (yellow dashed lines) were generated by using MARCM and examined 6 days after clone induction. Ovaries were stained with anti-GFP, anti-βPS and DAPI. High magnification views are shown in the right panels (B’, B’’, C’, D’, and D’’). StC nuclei are indicated by white arrowheads. (A, B) The distribution and level of βPS in *Inx2* mutant follicle cells at stages 2 to 3 (A), and stage 7 (B) were similar to that of GFP-negative adjacent control cells. (C, C’) In *FRT19A* control, βPS was barely detected in StCs. (D) *Inx2* mutant StCs failed to flatten. (D’) The intensity of βPS immunofluorescent staining signal in *Inx2* mutant StCs was significantly higher than that in the adjacent GFP-negative control cells. High levels of βPS were accumulated at the apical and lateral membranes of *Inx2* mutant StCs. (D’’) The distribution and level of βPS immunofluorescent staining signal in *Inx2* mutant posterior follicle cells were similar to that of the adjacent GFP-negative adjacent control cells. (E, E’) Surface view of follicle cells at stage 9. *Inx2* mutant StCs failed to flatten. The intensity of βPS immunofluorescent staining signal in *Inx2* mutant StCs, but not those posterior follicle cells, was significantly higher than that in the adjacent GFP-negative control cells. Length of the scale bar is 20 μm.

During oogenesis, the composition of integrins surrounding follicle cells changes dynamically (Dinkins *et al.* 2008; Delon and Brown 2009). Integrins are required for the morphogenesis from cuboidal to columnar shape of posterior follicle cells (Ng *et al.* 2016). We examined the distribution of the β subunit βPS in follicle cells during oogenesis. Single slice of confocal images were used for image analysis. βPS was detected on the apical domain of follicle cells prior to stage 6 (Supplementary Figure S6, A and B). From stages 7 to 8, βPS was detected on the apical-lateral domain of follicle cells (Supplementary Figure S6C). At stage 10, βPS is down-regulated in StCs and relocated to the basal domain in the posterior columnar follicle cells (Supplementary Figure S6D). We, therefore, speculated that βPS might play a role downstream of Inx2 in modulating StC morphogenesis. Prior to stage 9, the distribution and level of βPS in *Inx2* mutant follicle cells were the same as that of the neighboring GFP-negative control cells (Figure 4, A, B-B’’). At stage 9, the level of βPS was significantly higher in *Inx2* mutant StCs than that of the *FRT19A* control StCs or the neighboring GFP-negative control cells (Figure 4, C, C’, D, and D’, Supplementary Figure S5). βPS was accumulated on the apical and lateral membrane of *Inx2* mutant StCs (Figure 4D’). Furthermore, increase of βPS was observed specifically in *Inx2* mutant StCs but not in *Inx2* mutant posterior follicle cells (Supplementary Figure 4D’’). By using confocal microscopy to examine the surface plane the egg chamber, the level of βPS in *Inx2* mutant StCs was significantly higher than that of the neighboring GFP-negative control cells (Figure 4, E and E’). Our result demonstrates that *Inx2* deficiency leads to an increase of βPS specifically in StCs during morphogenesis.

### Genetic interaction between βPS and Inx2 in regulating StC morphogenesis

**Figure 5:**
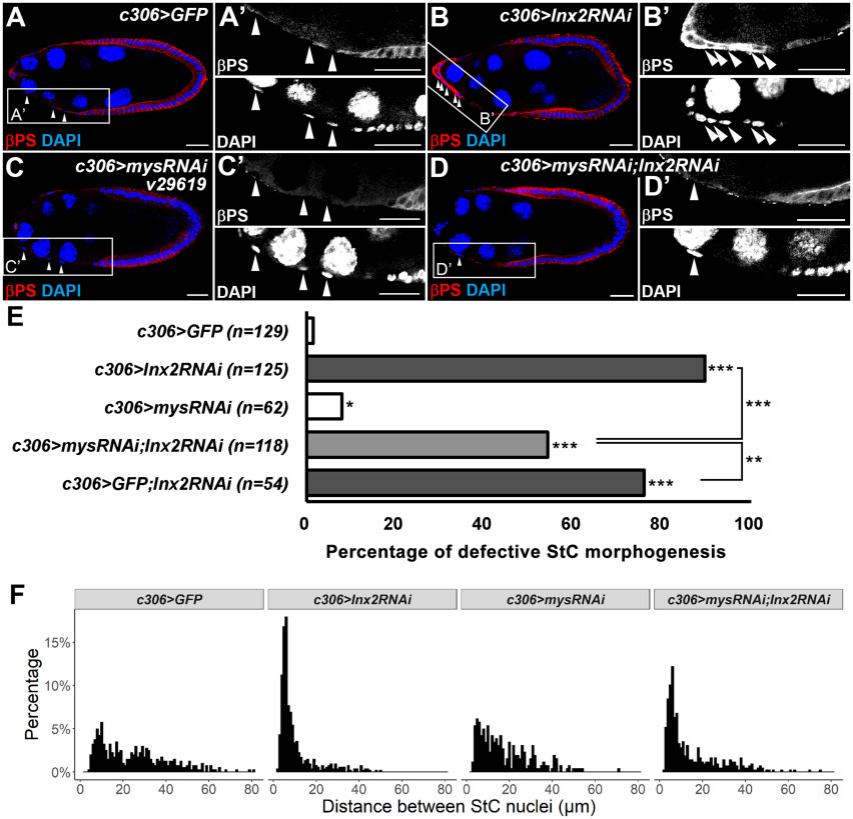
βPS acts downstream of Inx2 in modulating StC morphogenesis. Egg chambers at stage 9 were selected and oriented as anterior to the left. Newly eclosed flies were grown at 29°C for 6 days before dissection. Ovaries were stained with anti-βPS and DAPI. High magnification views were shown in the right panels (A’–D’). StC nuclei are indicated by white arrowheads. (A) *UAS-GFP* driven by *c306-GAL4* was used as a control. StCs flattened and nuclei were separated from one another. (B) Knockdown of *Inx2* attenuated StC flattening and led to a dramatic increase of βPS in StCs. (C) Knockdown of *mys* reduced the level of βPS in follicle cells at anterior and posterior ends of the egg chamber. StCs flattened and nuclei were separated from one another. (D) StCs flattened when *mys* and *Inx2* were knocked down simultaneously. The level of βPS in follicle cells at the anterior and posterior ends of the egg chamber was reduced. Length of the scale bar is 20 μm. (E) Quantitative analysis of the percentage of egg chambers with attenuated StC flattening by Fisher’s exact test of independence and post- hoc test (*P <* *0.05; **P  <* *0.01; ***P  <* *0.001). Knockdown of *Inx2* attenuated StC morphogenesis. Knockdown of *mys* partially rescued StC flattening defect in *Inx2* knockdown StCs. (F) Histograms of distance between adjacent StC nuclei. *c306>GFP* (45 egg chambers; 402 StC nuclei distance measured), *c306>Inx2RNAi* (38 egg chambers; 536 StC nuclei distance measured), *c306>mysRNAi* (35 egg chambers; 261 StC nuclei distance measured), and *c306>mysRNAi; Inx2RNAi* (49 egg chambers; 427 StC nuclei distance measured). The interval of each bar is 1 μm.

If excessive βPS in *Inx2* loss-of-function follicle cells attenuates StC morphogenesis, reduction of βPS may rescue the morphogenetic defect. We used *c306-GAL4* to drive shRNA expression. UAS-*GFP* was used as a control. In the control group at stage 9, StCs flattened and their cell nuclei were sparsely distributed (Figure 5, A and A’). Consistent with Figure 4D, knockdown of *Inx2* led to defective StC morphogenesis and an increase of βPS (Figure 5, B and B’). When *mys* was knocked down, StCs flattened normally at stage 9 (Figure 5, C and C’). The level of βPS in follicle cells at both anterior and posterior parts of the egg chamber was significantly reduced, demonstrating the efficiency of *mys* knockdown (Figure 5, C and C’). When *Inx2* and *mys* were knocked down simultaneously, flattened StCs were observed in many egg chambers (Figure 5, D and D’). Quantification analysis showed that knockdown of *mys* and *Inx2* significantly reduced the percentage of egg chambers containing aberrant StCs in comparison with knockdown of *Inx2* alone (Figure 5E), suggesting a partial rescue. To further quantify the phenotype of StC morphogenesis, we plotted the distance between adjacent StC nuclei as histograms (Figure 5F). The median for *c306>GFP, c306>Inx2RNAi, c306>mysRNAi*, and *c306>mysRNAi; Inx2RNAi* is 22.6, 6.5, 14.3, and 8.7 μm, respectively. In comparison with the distribution of *c306>GFP* control, the distribution of *c306>Inx2RNAi* dramatically shifted to the left (Figure 5F). The distribution and peaks of *c306>Inx2RNAi; mysRNAi* shifted to the right in comparison with the distribution of *c306>Inx2RNAi* (Figure 5F). To rule out the possibility that the partial rescue in Figure 5D has resulted from dilution of *c306-GAL4*, we used *c306-GAL4* to drive expression of both *GFP* and *Inx2* shRNA. Knockdown of *mys* and *Inx2* significantly reduced the percentage of egg chambers containing aberrant StCs in comparison with knockdown of *Inx2* and expression of GFP simultaneously (Figure 5E and Supplementary Figure S7). This result suggests that accumulation of βPS in *Inx2* loss-of-function cells may contribute to the attenuation of StC morphogenesis.

### Down-regulation of βPS induces early StCs morphogenesis

**Figure 6:**
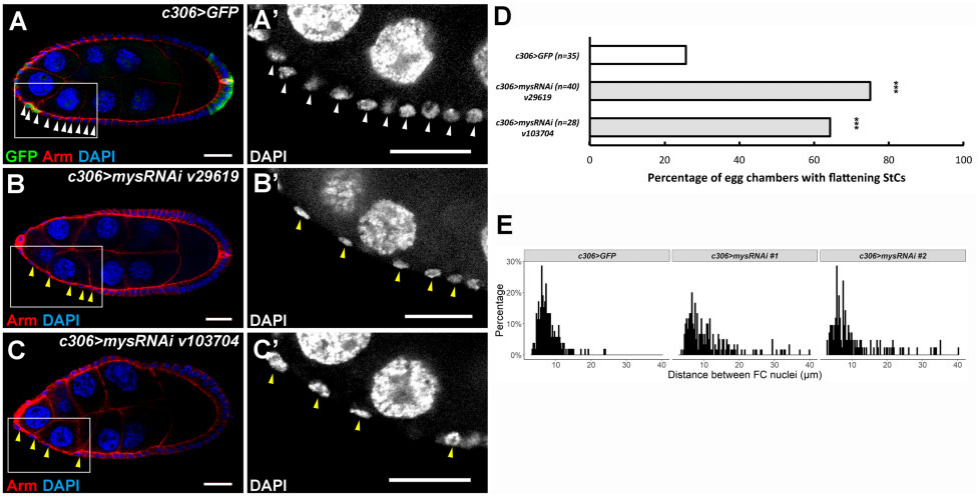
Reduction of βPS induces early StC morphogenesis. Newly eclosed flies were grown at 29°C for 6 days before dissection. Ovaries were stained with anti-Arm and DAPI. Egg chambers at stage 8 with the ratio of the oocyte to the egg chambers at 21–30% were selected and oriented as anterior to the left. High magnification views were shown in right panels (A’, B’, C’). StC nuclei are indicated by arrowheads. (A) In control group, follicle cell nuclei remained evenly distributed. (B, C) Knockdown of *mys* with two different RNAi lines led to early onset of StC flattening. StC nuclei were sparsely distributed. (D) Quantitative analysis of percentage of egg chambers with premature StC morphogenesis by Chi-squared test. Egg chambers with standard deviation (SD) of follicle cell distances>3 µm were categorized as egg chambers with flattening StCs. Knockdown of *mys* with two different RNAi lines led to early onset of StC flattening. ***P <* *0.001. Length of the scale bar is 20 μm. (E) Histograms of distance between adjacent StC nuclei. For each egg chamber, follicle cell nuclei distance of six adjacent cells were measured. *c306>GFP* (35 egg chambers), *c306>mysRNAi#1 v29619* (40 egg chambers), *c306>mysRNAi#2 v103704* (28 egg chambers). The interval of each bar is 1 μm.

If abnormal accumulation of βPS attenuates StC morphogenesis, reduction of βPS may induce StC flattening. To test this hypothesis, we used *c306-GAL4* to drive shRNA expression. Two *mys* RNAi lines with different shRNA targeting sequences were used. Egg chambers with oocyte occupying 21–30% of the egg chamber length were selected when most StCs in the control group remain cuboidal shape and the cell nuclei were tightly distributed (Figure 6, A and A’). More egg chambers in *mys* knockdown groups contained flattened StCs at this stage comparing with that of the control (Figure 6, B, B’, C, and C’). Quantitative analysis demonstrated that StC morphogenesis was observed in 25.7% of the egg chambers in *c306>GFP* control; 75 and 64.3% of the egg chambers in *c306>mysRNAi v29619* and *v103704*, respectively. To further demonstrate the phenotype of StC morphogenesis, we plotted the distance between adjacent follicle cell nuclei as histograms (Figure 6E). Six of the most anterior cell nuclei were selected for each egg chamber. The mean ± standard deviation (SD) for *c306>GFP, c306>mysRNAi v29619* and *v103704* is 6.9 ± 2.86, 8.93 ± 6.10, and 8.65 ± 6.33 μm, respectively. In addition to the increase of means in both *mys* knockdown groups, the SD for both *mys* knockdown groups was higher than that of *c306>GFP* control group, suggesting a more spread distribution of the distance between adjacent follicle cell nuclei when *mys* was knocked down (Figure 6E). Because βPS was down-regulated at stage 9 during StC morphogenesis (Figure 4C, Supplementary Figure S6), our data suggest that down-regulation of βPS may contribute to StC flattening.

### Inx2 regulates microtubules during StC morphogenesis

**Figure 7:**
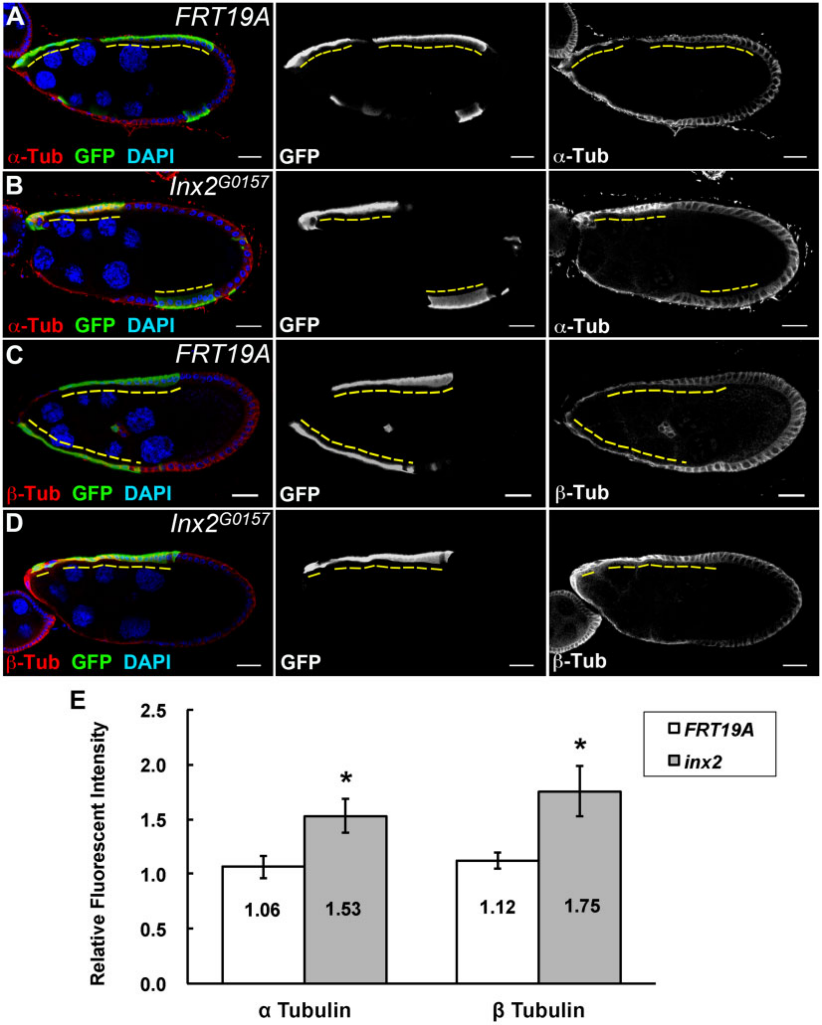
Inx2 regulates the level of microtubules during StC morphogenesis. Egg chambers at stage 9 were selected and oriented as anterior to the left. GFP-positive *FRT19A* and *Inx2^G0157^* mutant clones (yellow dashed lines) were generated by using MARCM and examined 6 days after clone induction. Ovaries were stained with anti-GFP (A-D), anti-α Tubulin (A, B), anti-β Tubulin (C, D), and DAPI. (A, C) Low levels of α and β Tubulins were detected in *FRT19A* control StCs. (B, D) High level of α and β Tubulins were concentrated at the lateral domain in *Inx2* mutant StCs comparing with that of adjacent GFP-negative control cells. Length of the scale bar is 20 µm. (E) The ratio of α or β Tubulin immunofluorescent intensities of GFP-positive *FRT19A* StCs to the adjacent GFP-negative StCs are close to 1.0. The ratio of α or β Tubulin immunofluorescent intensities of GFP-positive *Inx2* mutant StCs to adjacent GFP-negative StCs are higher than 1.0. Bar graph is shown as mean ± SEM. *P  <* *0.05 determined by using Student *t*-test.

Microtubules are critical for integrin trafficking (Seetharaman and Etienne-Manneville 2019). A recent study demonstrates that the level of microtubules was significantly decreased in *Inx2* mutant border cells (Miao *et al.* 2020), so we examined whether microtubules were affected in *Inx2* mutant StCs. In *FRT19A* control clones at stage 9, both α and β Tubulins were detected on the lateral of follicle cells (Figure 7, A and C). The immunofluorescent signals of α and β Tubulins were both increased in GFP-positive *Inx2* mutant cells comparing with the neighboring GFP-negative control cells (Figure 7, B and D). The fluorescent intensities of α and β Tubulins were significantly increased in *Inx2* mutant cells comparing with that in *FRT19A* control cells (Figure 7E). Thus, Inx2 may modulate the level of microtubules in StCs, which might be the mechanism for Inx2 to regulate the level of βPS. Alternatively, Inx2 may modulate microtubules and βPS in parallel. We further examined whether there was direct interaction between Inx2 and Tubulins by using PLA. No evidence of direct interaction between Inx2 and α Tubulin was observed based on PLA (Supplementary Figure S8, A, B, and E). Neither did we find evidence of direct interaction between Inx2 and βPS (Supplementary Figure S8, C, D, and E). Thus, Inx2 might regulate Inx2 and βPS through indirect interaction or other mechanisms.

## Discussion

In this study, we demonstrated that Inx2 is critical for the cuboidal-to-squamous transition of StCs in the *Drosophila* ovary. Blocking of gap junction functions did not interfere with StC morphogenesis, suggesting that Inx2 might modulate StC flattening through a gap junction-independent mechanism. We observe increases of βPS, DE-cad, and microtubules in *Inx2* mutant cells, which may contribute directly or indirectly to the morphogenetic defect of *Inx2* mutant StCs. Furthermore, reduction of *mys* partially rescued the morphogenetic defect of *Inx2* deficient StCs; reduction of *mys* alone induced early StC flattening. These data suggest that βPS may act downstream of Inx2 in modulating StC morphogenesis.

Among four *Inx*s expressed in follicle cells, only *Inx2* is required for StC morphogenesis (Figure 1). A previous study has shown that both *Inx2* and *Inx3* are required for epithelial organization and apical-basal polarity in embryonic epidermis. Knockdown of *Inx2* leads to mis-localization of Inx3, and vice versa, suggesting that Inx2 and Inx3 may form heteromeric channels (Lehmann *et al.* 2006). Furthermore, Inx2 regulates cell polarity through interaction with DE-cad and Arm in embryonic epidermis (Bauer *et al.* 2004). In our results, knockdown of *Inx3* does not affect StC morphogenesis (Figure 1D), suggesting Inx2 may not function together with Inx3 in StCs. In addition, cell polarity of *Inx2* loss-of-function follicle cells is not dramatically changed based on the correct distribution of DE-cad and Arm (Figure 3), suggesting that Inx2 regulates StC morphogenesis through a novel mechanism.

The TGF-β pathway has been shown to promote StC morphogenesis through remodeling of adherens junctions and cytoskeletons (Brigaud *et al.* 2015). *Dad-lacZ* is a well-accepted reporter reflecting the activity of the TGF-β pathway (Casanueva and Ferguson 2004). We demonstrate that *Dad-lacZ* was up-regulated in *Inx2* mutant follicle cells (Supplementary Figure S4), suggesting that Inx2 may negatively regulate the TGF-β pathway. Since the previous report demonstrates that activation of the TGF-β pathway promotes StC morphogenesis, we ruled out the possibility that Inx2 promotes StC morphogenesis through inhibiting the TGF-β pathway. As a transcriptional target of the TGF-β pathway, *Dad* encodes for the *Drosophila* homolog of inhibitory Smad (I-Smad), which antagonizes the receptor-regulated Smads and inhibits the TGF-β pathway activity (Tsuneizumi *et al.* 1997; Li *et al.* 2017). Therefore, it remains possible that by up-regulating *Dad*, Inx2 inhibits the TGF-β pathway, leading to attenuation of StC morphogenesis.

Integrins play various roles during *Drosophila* oogenesis, including FSC maintenance (O'reilly *et al.* 2008), follicle cell differentiation (Gomez-Lamarca *et al.* 2014), anterior–posterior polarity of the egg chamber (Diaz De La Loza *et al.* 2017), border cell migration (Dinkins *et al.* 2008; Llense and Martin-Blanco 2008), and elimination of the nurse cells (Timmons *et al.* 2017). Previous studies reported that knockdown of *mys* and reduction of Jun N-terminal kinase (JNK) signaling activity simultaneously, but not knockdown of *mys* alone, disrupt border cell cluster integrity and attenuates migration (Dinkins *et al.* 2008; Llense and Martin-Blanco 2008). In addition, a recent study demonstrates that stiffness of the basement membrane signals through integrins to modulate egg chamber elongation and StC morphogenesis (Chlasta *et al.* 2017). Here, we show that reduction of βPS alone induces early StC flattening. It would be interesting to further investigate how integrin signaling modulates StC morphogenesis.

We showed that blocking of gap junction functions with carbenoxolone attenuated the activity of the JAK/STAT pathway but not StC morphogenesis. These results suggest that Inx2 may modulate StC morphogenesis independently of its gap junction activity. However, specificity and efficiency are always concerns for pharmacological approaches. While we cultured ovaries *ex vivo* for 6 hours, it remains possible that carbenoxolone did not block gap junction functions completely in this short period of time. While the underlying molecular mechanism of Inx2 in modulating StC morphogenesis remains unclear, one possibility is that gap junction proteins serve as scaffolds for protein–protein interaction. In mammals, Cx43 is a predominant Connexin in myocardium and epithelial tissues. Cx43 interacts with structural proteins such as cytoskeletal proteins, proteins related to trafficking or proteins in junctional complexes (Sorgen *et al.* 2018). Here, we observe an increase of microtubules in *Inx2* mutant StCs (Figure 7). It is possible that the defect of microtubules in *Inx2* deficient StCs leads to attenuation of protein trafficking and abnormal accumulation of βPS and DE-cad, therefore interfering with StC morphogenesis. However, no evidence of direct interaction between Inx2 and tubulins was found based on PLA (Supplementary Figure S8). Androcam (Acam) and CG4942 are reported to physically interact with Inx2 based on two-hybrid system (Giot *et al.* 2003). Acam is a testis-specific light chain for myosin VI (Frank *et al.* 2006). CG4942 is a membrane insertase located in the mitochondria. Neither of them is a promising candidate for StC morphogenesis. Thus, identification of proteins physically interacting with Inx2 may help us to further understand the molecular mechanism underlying StC morphogenesis.

A recent study demonstrates that Connexin 30 (Cx30) sets the orientation of astroglial motile protrusions via modulating the laminin/integrin/Cdc42 polarity pathway in a cell culture model. Over-expression of Cx30 disrupts cell polarity and reduces the level of laminin and integrins in cultured astrocytes. Importantly, over-expression of a deficient Cx30 that cannot form gap junctions still disrupts cell polarity, suggesting that Cx30-mediated regulation of astrocyte polarity does not require gap junction functions (Ghezali *et al.* 2018). This finding is similar to our results in StCs of the *Drosophila* oavry*.* How Cx30 regulates the level of laminin and integrins in cultured astrocytes remains unclear. We and others demonstrate that Inx2 regulates microtubules in both border cells and StCs in the *Drosophila* ovary in gap junction-independent manners (Miao *et al.* 2020). Interaction between gap junction proteins and integrins may be an evolutionarily conserved mechanism in regulating various cellular behaviors.
